# Editorial: Attachment theory in educational psychology

**DOI:** 10.3389/fpsyg.2026.1898412

**Published:** 2026-06-24

**Authors:** Laura E. Prino, Rosalba Morese, Matteo A. Fabris

**Affiliations:** 1 Department of Philosophy and Education Sciences, Università degli Studi di Torino, Turin, Italy; 2 Università della Svizzera Italiana, Lugano, Switzerland; 3 Department of Psychology, Università degli Studi di Torino, Turin, Italy

**Keywords:** adolescents, attachment, children, educational research, emotional security, schools, students, teachers

Attachment theory has profoundly influenced the understanding of human development by emphasizing the role of close relationships in fostering emotional security, exploration, and adaptation across the lifespan. Since Bowlby's (1969) original formulation and Ainsworth's empirical contributions (Ainsworth et al., 1978), attachment theory has extended beyond the family context, inspiring research on relational experiences within educational settings. Schools are not merely places for the transmission of cognitive knowledge but are relational environments where interactions with teachers, peers, and significant adults influence students' emotional security, wellbeing, motivation, and sense of belonging (Bergin and Bergin, 2009; Fabris et al., 2022, 2024).

Over the years, attachment-informed educational research has shown that school relationships can serve as a “secure base” and a “safe haven,” supporting wellbeing, motivation, emotion regulation, and psychological adjustment (Prino et al., 2023). The growing attention to teacher–student relationships, social-emotional learning, loneliness, and trauma-informed educational practices reflects the continuing relevance of attachment theory for educational psychology (Butler and Sultana, 2025; Scarzello and Prino, 2025).

The aim of this Research Topic, “*Attachment theory in educational psychology*,” was to deepen current knowledge of the contribution of attachment theory to educational processes and developmental outcomes across different educational contexts and developmental stages. Although the contributions adopt different theoretical perspectives and methodologies, they share a common assumption: educational experiences are deeply relational, and relational security is a fundamental condition for learning, wellbeing, and adjustment.

Several contributions emphasize the importance of emotionally supportive relationships in promoting students' engagement and academic achievement. In a study of teacher education students, Ma examines how social-emotional competence contributes to classroom participation. The findings highlight that participation is influenced not only by cognitive abilities or individual motivation, but also by students' capacity to manage the interpersonal and emotional aspects of classroom life. The centrality of supportive educational relationships also emerges in the work of Wang et al., who investigate the link between teacher caring behavior and English learning achievement. Their model shows that teacher care supports learning both directly and indirectly through emotional and motivational processes. Similarly, Zhang, Yu et al. conceptualize career-related teacher support as a “secure base” that promotes career adaptability and future work self-salience among university students. The contribution by Hormann et al. further illustrates the importance of teacher–child interactions for socio-emotional development. Taken together, these studies describe educational relationships as developmental contexts in which emotional security fosters curiosity, participation, learning, and identity construction.

A second group of studies examines self-regulation, emotional adjustment, and academic achievement during adolescence. Sun and Zhang investigate the relationship between self-regulated learning and academic achievement, focusing on the mediating role of academic emotions. Their findings highlight the connection between emotional processes and learning regulation, confirming that successful school adjustment depends not only on cognitive strategies but also on students' emotional experiences. From a complementary perspective, Zhang, Zhao et al. study how parenting styles affect adolescents' test anxiety through the mediating role of academic grit. Their results show that relational experiences within the family context continue to shape students' emotional responses to academic challenges and their academic grit and perseverance.

Together, these studies reinforce a core assumption of attachment theory: internal working models influence how individuals regulate emotions, cope with stress, and approach achievement-related challenges. Academic achievement, therefore, cannot be interpreted solely in cognitive terms but should be understood within broader relational and emotional systems.

Another central theme concerns students' social connectedness and sense of belonging within school contexts. Zheng et al. address loneliness in schools, showing that teachers view relational support and inclusive classroom practices as essential tools for addressing student loneliness, despite structural barriers in educational settings. From an attachment-informed perspective, loneliness signals unmet relational needs, making schools crucial spaces for promoting connectedness and emotional security. Fan et al. also explore relational processes in peer contexts, examining how group dynamics influence children's behavioral choices in sports. In a related vein, Liu and Shi investigate motivation in university volunteer teaching programs, highlighting how social recognition, organizational support, and a sense of belonging are key elements in sustaining volunteers' engagement and wellbeing.

The attachment framework also provides useful insights into adults' wellbeing within educational contexts. Zhao et al. examine Head Start educators' professional wellbeing and turnover intentions, focusing on the role of perceived workplace discrimination. Their findings highlight the relational and organizational conditions that sustain or undermine educators' wellbeing, with important implications for educational climate and students' wellbeing.

Overall, the studies included in this Research Topic confirm the value of attachment theory for educational psychology. Across different developmental stages and educational domains, the contributions converge in emphasizing that learning and development are inseparably linked to relational experiences. Teacher support, emotional attunement, social belonging, family relationships, and institutional climate all contribute to sustaining students' wellbeing, engagement, and adjustment.

This Research Topic demonstrates how attachment theory continues to provide a particularly fruitful framework for understanding the contemporary challenges of educational psychology, from promoting mental health and social inclusion to creating educational contexts that are more equitable, secure, and relationally meaningful.
